# The immunological personality of close relatives of SLE patients

**DOI:** 10.1177/0961203317707826

**Published:** 2017-05-23

**Authors:** M R Salaman, D A Isenberg

**Affiliations:** 1 Department of Medicine, St Mary's Campus, Imperial College, London, UK; 2 Centre for Rheumatology, University College London, London, UK

**Keywords:** Relatives of lupus patients, immunological abnormalities, immunoglobulin regulation, NK and NKT cells, lupus pathogenesis

## Abstract

Immunological abnormalities seen in relatives of patients with autoimmune disorders can be useful in understanding the pathogenesis of the disease since, unlike in patients, they cannot result from the disease process or drug treatment. In this article we present a brief overview of our studies of the basic immunological status of close relatives of SLE patients. We looked at blood levels of IgG, IgM and antibodies to double-stranded DNA, as well as at NK cell numbers and cytotoxic activity and the levels of NKT, B and T cells. As many as 60% of relatives showed one or more abnormalities in these assays. Most notably there were increased levels of IgG in male and female relatives and a reduction of IgM in females. IgG correlated inversely with NKT cell numbers adding strength to the concept that the presence of IgG autoantibodies in patients is due to impaired regulation by NKT cells. IgM, on the other hand, correlated inversely with NK cells which may thus have a role in bringing about the reduced IgM seen in some patients. Immunological abnormalities were found to be more often associated with parents and offspring of patients than with their siblings, pointing to the involvement of environmental or epigenetic influences in lupus pathogenesis.

Although it is well established that autoantibodies are present in some close relatives of SLE patients,^1^ little attention has been paid to their basic immunological status. Some years ago we carried out two studies exploring this subject, the data from which are distributed over several publications. It may be helpful now to bring the findings together in a brief overview, incorporating some more recent information.

In our studies of healthy male and female first-degree relatives of lupus patients we frequently observed abnormal values of a number of immunological parameters in comparison to controls.^2–4^ Such abnormalities are noteworthy since unlike those in patients they cannot be the result of the disease process or drug treatment, pointing therefore to genetic or environmental factors in disease pathogenesis.

These abnormalities were observed in several IgG and IgM assays including those for plasma immunoglobulin and antibodies to double-stranded DNA. They were also observed in assays for natural killer (NK) cell cytotoxic activity and blood levels of T, B, NK and NKT cells. Recently we have defined a more stringent criterion for an abnormal value and provided an analysis showing that such abnormalities are more likely to be found in parents and offspring of patients than in siblings.^5^ Overall nearly 60% of relatives showed at least one abnormal value. Where relatives had more than one abnormal value, no particular grouping of activities was discernible apart from that previously described involving B cells, T cells and IgG.^5^

The existence of such abnormal values does not necessarily reflect a significant difference between relative groups and healthy controls. There was, however, a significant reduction of plasma IgM in female relatives (*p* < 0.01) and significant increase in IgG in the combined (male and female) group (*p* < 0.05).^4^ There was also an increased capacity in a combined relative group to produce IgG on stimulation of blood lymphocytes with pokeweed mitogen (*p* < 0.05).^2^ There was no significant change in the numbers of the different lymphocytes (cells per ml of blood), but when expressed as a percentage of total lymphocytes there was a significant increase for B cells in the female relative group (*p* < 0.01).^3^

To get an indication whether the altered values of IgG and IgM could be linked to the blood level of the four different types of lymphocyte we have looked at correlations within male and female relative groups (ref.4 and further analysis). There was for female relatives a highly significant negative correlation between NK cell numbers and IgM (*r* = −0.54, *p* < 0.005) and a weaker negative correlation between B cells and IgM (−0.40, *p* < 0.05). There was no IgM correlation in this group with T or NKT cell numbers, but expressed as cell frequency there was a positive correlation for %T cells (0.58, *p* < 0.001). The above-mentioned NK correlation persisted strongly expressed as frequency (−0.48, *p* < 0.01). Remarkably, with regard to IgG, the only correlation was with NKT cells. There was a strong negative correlation with NKT cell numbers in the male relative group (−0.69, *p* < 0.01) while a similar relationship in the female relatives approached significance (−0.34, *p* < 0.08). As NKT cell frequency the male group retained the same level of correlation (−0.70, *p* < 0.01) and the female group now showed significance (−0.39, *p* < 0.05). Significant correlations were not seen in the female controls. There were too few male controls for such an analysis, but again no correlations were apparent.

The depressed IgM levels seen in female relatives are of interest in relation to the low levels that were seen in some 17% of SLE patients.^4^ It has been reported that patient levels of IgM decline with increasing duration of disease.^6,7^ However, as many as 6% of first-degree relatives of SLE patients may develop the disease,^8^ so that the finding of low blood IgM levels in female relatives suggests that the low level seen in patients is at least in part a primary genetic or environmental manifestation of their disease. Moreover, our correlation data suggest that NK cells, and possibly also B cells themselves, play a regulatory role in bringing about the reduced output of IgM in these relatives. This phenomenon might be analogous to that described in mice in which gamma-interferon-producing NK cells inhibit IgM production.^9^ On the other hand, patients, in whom no comparable correlation was established, paradoxically have a low level and activity of NK cells.^3^ Correlations involving percentage of cell types (e.g., %T cells) but not evident for cell numbers must be interpreted with particular caution since percentage data will be influenced by changes in the numbers of other cell types (NK and B cells).

A much clearer picture emerges from our observations on IgG.^4^ Raised levels of IgG were found in both male and female relatives mirroring the situation in SLE patients. Inverse correlations were found in relatives between IgG and NKT cell numbers and no other lymphocyte type showed correlations. It seems likely therefore that the high levels of IgG in both patients and relatives is due at least in part to impaired regulation by NKT cells. It should be noted that the raised IgG in relatives will be largely non-autoimmune,^2^ while that in SLE includes high levels of pathogenic autoantibodies, additional factors of course being involved in patients.

We cannot say whether this proposed regulatory function of NKT cells applies to the invariant cells (iNKT) or to other types of NKT cell, but iNKT cells have been shown to be at a low level in several autoimmune diseases including SLE.^10,11^ Moreover, Wither et al.^12^ have obtained evidence that iNKT cells are similarly reduced in patient relatives. Such cells are strong cytokine producers and regulatory activity may well come about by cytokine action on the immune cells involved in IgG production. It may be significant that reduced levels of the regulatory cytokines TGF-β and IL-10 have been observed in relatives.^13^

In the study by Wither et al.^12^ abnormalities were demonstrated in first-degree relatives of SLE patients relating to the proportion of various B and T cell subsets and in the frequency of antinuclear antibody (ANA) positivity. In the case of certain B cell subtypes and of ANA the abnormalities were more pronounced in parents than in siblings, thus concurring with our findings for different types of relative.^5^ Further, their observation that parents of SLE patients are much more likely to suffer from other autoimmune diseases than siblings points to a wider significance for this asymmetry. It might be hoped that a definitive comparison of the risk of developing SLE when your sibling as opposed to your parent/offspring has the disease would come from a study such as that of Kuo et al.^14^ involving nearly 24 million subjects! A greater risk, by contrast, was reported for siblings. However, such studies have age-related problems; for example, affected parents may have died without inclusion in the study and younger people may not yet have shown symptoms of the disease.

A list of the most common abnormalities that we have observed in relatives of SLE patients is shown in Table 1. Also included are references to studies by other groups demonstrating the presence in relatives of autoantibodies, high levels of plasma alpha-interferon (IFN-α) and high or altered levels of other pro-inflammatory or regulatory cytokines/mediators. Our work was too short-term to find out whether the presence of abnormalities can be used as an early warning of possible transition to SLE. There is already, however, preliminary evidence that such an approach could be useful.^13,15^

**Table 1:** Immunological abnormalities in first-degree relatives of SLE patients

Biomarkers	Abnormality*	Comments
Plasma IgG	Raised^2,4^	Inverse correlation with blood NKT cells
Plasma IgM	Reduced (females)^4^	Inverse correlation with blood NK cells
Pokeweed mitogen-induced IgG	Raised^2^	High capacity for IgG production
B lymphocytes	Increased frequency in lymphocyte pool (females)^3^	
T lymphocytes	Activated state (females)^17^	Indirect evidence of T cell activation
Miscellaneous	Variable frequency of abnormalities among different relative types^5,12^	Suggestive of environmental or epigenetic influences in their inheritance
Autoantibodies	Presence of anti-DNA and other autoantibodies^1,12,13,15^	
Pro-inflammatory and regulatory cytokines/mediators	High or altered levels of IFN-α, IFN-induced and other proteins^13,16^	

* Abnormality observed in both male and female relatives unless specified.

In summary, both male and female first-degree relatives of SLE patients have a tendency to show abnormal values of standard immunological parameters. These data suggest an important role for NKT cells in regulation of IgG production in both relatives and patients and a possible similar role for NK cells in relation to IgM. The striking tendency of female relatives to show correlations between various immunological data^2^ is suggestive of a highly active, though not well regulated, immunological state in such relatives. In many SLE patients T cells are in an activated state and indirect evidence suggested this may also be true for female relatives.^17^ The asymmetric appearance of abnormalities in different types of relative is indicative of environmental or epigenetic influences in lupus pathogenesis and may provide clues as to their nature.^5^ Basic genetic mechanisms will of course also be involved and genome-wide studies have identified many genes including some concerned with lymphocyte development and activation.^18^ Matching these genes to the abnormal behaviour will be a task for the future.
